# Patient Engagement With a Game-Based Digital Therapeutic for the Treatment of Opioid Use Disorder: Protocol for a Randomized Controlled Open-Label, Decentralized Trial

**DOI:** 10.2196/32759

**Published:** 2022-01-26

**Authors:** Hilary Luderer, Lisa Chiodo, Amanda Wilson, Christina Brezing, Suky Martinez, Xiaorui Xiong, Robert Gerwien, Bruce Imbert, Mark Deeg, Yuri Maricich, Aimee Campbell

**Affiliations:** 1 Pear Therapeutics, Inc Boston, MA United States; 2 Addiction Research and Education Foundation/Clean Slate Florence, MA United States; 3 Columbia University New York, NY United States

**Keywords:** decentralized trial, game-based, gamification, prescription digital therapeutic, digital therapeutics, opioid use disorder, reSET-O, virtual trial, trial, game, therapy, opioid, drug, engagement, treatment, disorder, addiction, randomized controlled trial, mental health, symptom

## Abstract

**Background:**

Prescription digital therapeutics are software-based disease treatments that are regulated by the US Food and Drug Administration; the reSET-O prescription digital therapeutic was authorized in 2018 and delivers behavioral treatment for individuals receiving buprenorphine for opioid use disorder. Although reSET-O improves outcomes for individuals with opioid use disorder, most of the therapeutic content is delivered as narrative text. PEAR-008 is an investigational device based on reSET-O that uses an interactive, game-based platform to deliver similar therapeutic content designed to enhance patient engagement, which may further improve treatment outcomes.

**Objective:**

We aim to investigate how participants interact with the prescription digital therapeutic’s new content delivery format. Secondary objectives include evaluating treatment success, symptoms of co-occurring mental health disorders, recovery capital, and skill development.

**Methods:**

Due to the COVID-19 pandemic, this study was redesigned using a decentralized model because it was not possible to conduct medication initiation and study visits in person, as initially intended. A decentralized, randomized controlled trial design will be utilized to compare patient engagement with PEAR-008 and that with reSET-O using both subjective and objective assessments. The study population will consist of approximately 130 individuals with opioid use disorder (based on Diagnostic and Statistical Manual of Mental Disorders 5 criteria) who have recently started buprenorphine treatment for opioid use disorder. Participants will be virtually recruited and randomly assigned to receive either PEAR-008 or reSET-O. All study sessions will be virtual, and the duration of the study is 12 weeks. The primary outcome measure of engagement is operationalized as the number of active sessions per week with either PEAR-008 or reSET-O. (An active session is any session that contains some active participation in the app, such as navigating to a different screen, engaging with a learning module, or responding to a notification.) Subjective dimensions of engagement will be assessed with participant surveys. The hypothesis is that PEAR-008 will have significantly greater participant engagement than reSET-O.

**Results:**

As of February 2021, participant enrollment is ongoing.

**Conclusions:**

This randomized controlled trial will investigate if changing the delivery format and enhancing the content of a prescription digital therapeutic for opioid use disorder will affect how participants use and interact with the prescription digital therapeutic. The study design may serve as a useful model for conducting decentralized studies in this patient population.

**Trial Registration:**

ClinicalTrials.gov NCT04542642; https://clinicaltrials.gov/ct2/show/NCT04542642

**International Registered Report Identifier (IRRID):**

DERR1-10.2196/32759

## Introduction

### Background and Rationale

The United States is in the midst of an opioid overdose epidemic [1,2]. Underlying opioid use disorder is a key driver of this epidemic, and approximately 1.6 million people in the United States met criteria for opioid use disorder in 2019 [3]. Opioid use disorder is a chronic disease with a range of physical, psychological, and personal consequences, including high mortality. Opioids are particularly hazardous due to the rising prevalence of potent illicit opioids (predominantly fentanyl) with high risk of lethality [4]. During the COVID-19 pandemic, overdose deaths surged to an all-time high of 92,183 in the United States, driven primarily by synthetic opioids [1]. Maximizing the impact of effective therapies that are easily accessible during the pandemic and beyond is critical to helping individuals with opioid use disorder receive optimal and necessary care.

Pharmacologic treatments, such as US Food and Drug Administration (FDA)–approved medications (for example, buprenorphine, naltrexone, and methadone), are the first line of treatment for opioid use disorder, in conjunction with evidence-based behavioral therapies, but the majority of individuals in need of treatment (80% to 90% [2,5,6]) for substance use disorders do not receive care. There are a variety of contributing factors, including refusal to seek treatment, high cost of care, stigma associated with care, homogeneity of treatments offered, and lack of or limited access to treatment [7,8]. For individuals with opioid use disorder who do seek treatment, there is significant variability in quality and utilization of evidence-based therapies across providers [9,10]. Significant training, time, and clinical oversight are required to ensure proper face-to face delivery of behavioral therapy [11]. Digitizing and offering evidence-based therapies on mobile devices can standardize care, ease the burden on clinical staff, and expand access to behavioral treatment.

Prescription digital therapeutics are software-based treatments that have been evaluated for safety and effectiveness in randomized clinical trials and authorized by the FDA. Prescription digital therapeutics have the potential to safely expand access to evidence-based interventions because they are delivered on mobile devices and are prescribed and initiated by treatment providers.

Two prescription digital therapeutics are currently available—reSET and reSET-O—to deliver digitized behavioral therapy for substance use disorder and opioid use disorder, respectively [12,13]. Both prescription digital therapeutics deliver therapy modeled on the community reinforcement approach, which is an evidence-based treatment that promotes behavioral change [14]. Although studies indicate prescription digital therapeutics hold promise in treating substance use disorder and opioid use disorder [15-20], the current content delivery method used by these prescription digital therapeutics is largely didactic, with the majority of content delivered as narrative text. PEAR-008 is an investigational device that delivers therapeutic content similar to reSET-O via an interactive game-like environment designed to maximize patient engagement and satisfaction—factors that are critical in retaining patients in opioid use disorder treatment [21-26]. We hypothesize that the use of a more interactive and engaging platform to deliver similar therapeutic content will enhance patient engagement with a digital therapeutic.

## Methods

### Overview

We will compare reSET-O to PEAR-008 and evaluate objective differences in participant engagement with each digital therapeutic. Secondary outcomes include subjective differences in engagement, opioid use disorder treatment outcomes (ie, retention in treatment and abstinence from opioids), symptoms of common comorbid mental health conditions, including anxiety and depression, recovery supports (eg, recovery capital and resilience), and participant satisfaction with their assigned digital therapeutic. Exploratory aims include evaluating development of cognitive behavioral therapy skills and buprenorphine adherence.

### Study Design

This is a 2-arm, randomized controlled, open-label, outpatient-based study to be conducted virtually by 2 recruitment sites: the New York State Psychiatric Institute’s Substance Treatment and Research Services (STARS) program at Columbia University Irving Medical Center and the Addiction Research and Education Foundation (AREF). Study participants will be recruited from outpatient addiction specialty treatment programs and individual providers in the United States.

### Study Population and Sample Size

The trial will include 130 adults aged 18 to 60 years with opioid use disorder who are already receiving buprenorphine treatment, with no criteria regarding gender identity, race, or ethnicity.

Inclusion criteria are having the ability to provide informed consent, age 18 to 60 years, having adequate English proficiency, being within the first 120 days of starting buprenorphine, receiving buprenorphine pharmacotherapy under the care of a licensed health care provider and being willing to provide the name of the provider or practice, being capable of using common software apps on a smartphone, and having access to an internet-enabled smartphone that meets minimal operating system requirements for the duration of the study. Exclusion criteria are having a history of reSET-O use, having participated in user-testing of PEAR-008 or any investigational drug trials within 30 days of trial enrollment, or currently receiving methadone or naltrexone pharmacotherapy.

The sample size and power were based on the primary engagement outcome, determined by frequency of interaction with the intervention, percentage of module completion, and approval ratings. A sample size of 130, α=.05, and power=0.80 will allow for detection of a moderate effect size (*d*=0.50).

### Study Settings and Recruitment Procedures

All study sessions will be conducted virtually by study staff at 2 participating organizations: New York State Psychiatric Institute’s STARS and AREF.

Since its establishment in 1997, the STARS clinic of Columbia University has served as the center for clinical trial operations within the Division on Substance Use Disorders. Recruitment will be directed to individuals who live in New York, New Jersey, and Pennsylvania. Participants may be recruited via flyers in office spaces or intake packets (as allowed; flyers will be disseminated electronically to providers and opioid treatment programs) or via word of mouth by providers. In addition, potential participants may be referred to this study after screening procedures conducted in concurrent research studies taking place at STARS (eg, if they are ineligible for concurrent studies, they may be referred to this study for screening).

AREF is a research foundation that conducts and disseminates research related to addiction medicine to advance the science surrounding the treatment of individuals with a substance use disorder. AREF conducts research activities with patients recruited from a multistate network offering guideline-driven outpatient treatment with buprenorphine for individuals with opioid use disorder in a group practice setting. When possible, flyers will be posted in the waiting room at treatment locations (where patients are being seen face-to-face, which is dependent on local regulations related to COVID-19). Flyers will be included in patient packets that are emailed or distributed to new patients. Additional participants may also be recruited via online advertisements (eg, Craigslist).

Participant flyers will provide the URL for the study website as well as a QR code to access the website. On the study website individuals will find information about the study and a form to express interest in participating in the study. Data entered on the website are sent to an encrypted database housed by Formstack, a Health insurance Portability and Profitability Act–compliant platform and to the sites via PGP-encrypted emails.

### Randomization and Blinding

Following informed consent, the baseline assessment and confirmation of eligibility, participants will be randomly assigned to 1 of 2 treatment groups: reSET-O or PEAR-008. Randomization lists will be prepared by the study sponsor (Pear Therapeutics) prior to the start of the study. Randomization will be 1:1 and stratified by site and gender with a block size of 10. An electronic list of IDs, access codes, and credentials will be securely provided to the sites. There will be no blinding; this is an open-label study.

### Study Interventions

#### reSET-O

reSET-O is a prescription digital therapeutic for opioid use disorder delivered concurrently with standard buprenorphine treatment [13]. reSET-O delivers therapy in the form of a series of 67 interactive lessons via a patient-facing mobile software app. A typical therapy lesson comprises a behavioral therapy component and skill-building exercises. The therapeutic content is based on the community reinforcement approach, an intensive addiction-specific form of cognitive behavioral therapy that has been validated for opioid use disorder [14]. Therapy lesson content is delivered primarily via written text but may include videos, animations, and graphics. After most therapy lessons, the user undergoes fluency training, which is a method of questioning that has been demonstrated to promote learning and improve both short-term and long-term retention of material [27].

reSET-O also includes contingency management delivered via a virtual rewards wheel. Contingency management rewards are either virtual (thumbs up icon) or tangible rewards (gift card with value range US $5 to $100) that can be earned for completion of therapy lessons. Studies [28] have consistently demonstrated that contingency management interventions, particularly abstinence-based incentives, can support treatment and recovery in individuals with a wide range of substance use disorders. The odds of winning a tangible reward are 50% each time a reward is possible (wheel spin or mystery box), with higher-value rewards occurring less often. The odds of receiving a $100 gift card are 0.2%, whereas the odds of receiving a $5 gift card are 41.8%. The value of the rewards is commensurate with the amount of time users typically spend with the prescription digital therapeutic over the 12 weeks of treatment. Users are also prompted to report substance use, cravings, and triggers every 72 hours. A user can self-initiate reports of substance use, cravings, and triggers at any time. reSET-O contains an optional daily medication reminder that can be set to enhance adherence to buprenorphine for opioid use disorder.

#### PEAR-008

PEAR-008 is an investigational device with therapeutic content and a contingency management reward system that are similar to those in reSET-O; however, clinical content enhancements have been made, and the therapy has been reformatted as a game with mechanics designed to promote engagement. Content enhancements include use of person-centered language and lowering the reading level to make it more accessible. In PEAR-008, the reSET-O therapy lessons were divided into a tiered structure of shorter chapters with the intent of providing small, achievable goals to help keep users motivated and engaged. Each chapter has an associated quiz based on the fluency training approach.

**Figure 1:**
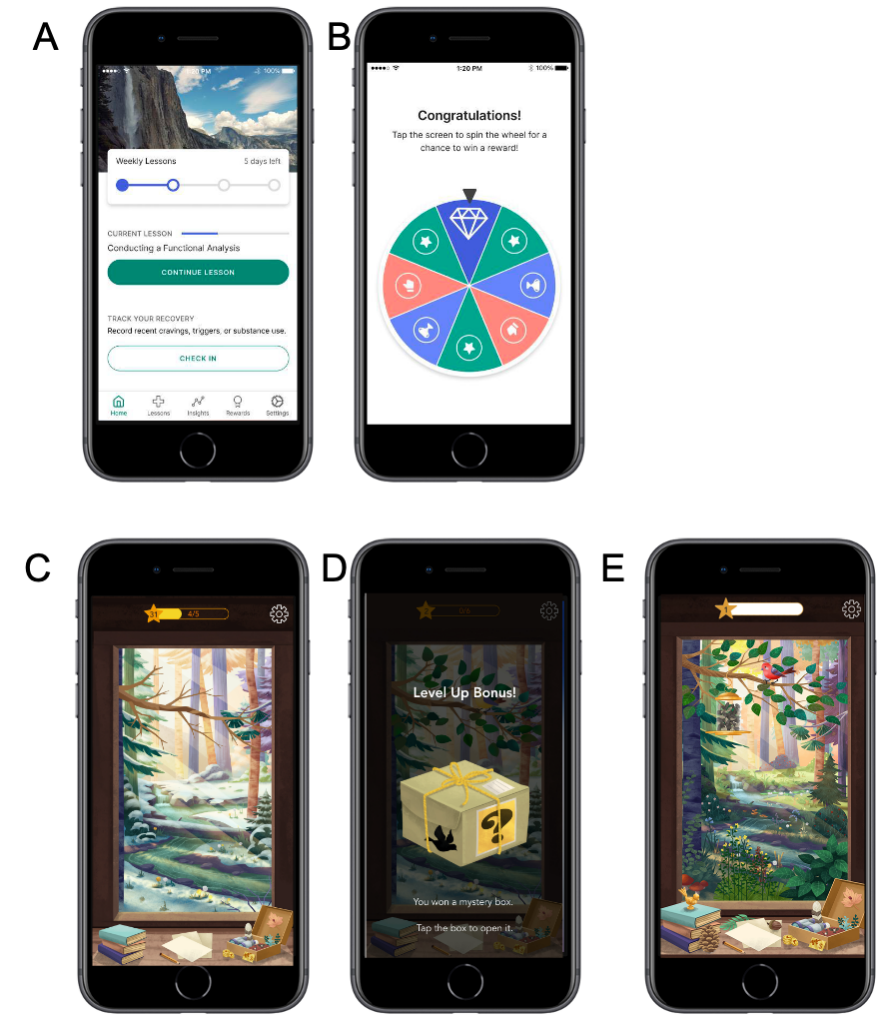
Screenshots from reSET-O (A) home screen and (B) contingency management rewards wheel screen, and PEAR-008 (C) home screen with winter scene, (D) contingency management rewards mystery box screen, and (E) home screen with summer scene. Content is accessed via the icons on the windowsill, lessons are represented by the stack of books icon, worksheets are represented by paper and pencil, and rewards are represented by the treasure box.

The PEAR-008 home screen shows a nature scene that transitions from winter to summer as the patient progresses through therapy (Figure 1). The game economy of PEAR-008 is variable, consisting of the ability to earn stars that unlock virtual rewards such as birds and home-screen upgrades, as well as Mystery Boxes. The Mystery Boxes replace the contingency management rewards wheel in reSET-O and contain either virtual (stars) or tangible rewards (gift card with value ranges from $5 to $100) with the same odds of winning a tangible reward at each level of value as those in reSET-O. Similar to reSET-O users, PEAR-008 users earn the opportunity for contingency management rewards by completing fluency training quizzes (ie, therapy lessons). Engagement is also incentivized: a user can earn rewards by repeating a chapter and by engaging at least once daily with PEAR-008. The user is required to complete a daily check-in the first time they open the app each day. The check-in consists of a series of questions related to substance use, medication use, general recovery status, and recovery-specific questions regarding cravings, triggers, and problems the user is managing.

### Study Procedures

#### Overview

All study visits will be conducted remotely. Each prospective participant will complete a short screening assessment, and if basic eligibility is confirmed, complete informed consent procedures and sign an electronic informed consent form prior to completing the baseline assessment. After baseline assessments, participants will be randomly assigned to either reSET-O or PEAR-008 groups. Study staff will assist each participant with installation of their assigned digital therapeutic on their mobile device. Staff will provide training on the app’s use. Study participants will be enrolled for 12 weeks, with weekly virtual sessions during weeks 1-8 and a final visit at week 12, to evaluate the impact of reSET-O and PEAR-008 on participant engagement and treatment outcomes. Participants will also complete slightly longer assessments at weeks 4 and 8.

Weekly study assessments completed during the first 8 weeks of treatment include saliva drug screening (participants will receive drug screening equipment on a regular basis by mail; urine drug screen results may be retrieved from the electronic medical record for AREF participants), timeline followback [29], and adverse event reporting. Additional self-report assessments will be delivered at week 4 and week 8.

Participants will attend a virtual end of treatment study session at 12 weeks to complete the following: saliva drug screening, timeline followback, and adverse event reporting assessment. Additional measures will be assessed at the 12-week follow-up visit (Table 1).

**Table 1:** Study objectives and endpoints.

Objective			Endpoint
**Primary**			
	To evaluate participant engagement with PEAR-008 compared to that with reSET-O. The hypothesis is that PEAR-008 group will have significantly greater participant engagement than the reSET-O group.	Number of active sessions with PEAR-008 or reSET-O per week	
**Secondary**			
	To evaluate the impact of PEAR-008 compared to reSET-O on treatment retention	Time to dropout (last contact with a participant)	
	To evaluate the impact of PEAR-008 compared to reSET-O on abstinence from illicit opioids	Abstinence will be defined as abstinence on patient self-reports (via timeline followback) and the absence of nonbuprenorphine opioids on saliva drug screens. Abstinence (binary outcome: yes or no) will be determined 9 times, weekly during weeks 1-8 and at week 12.	
	To evaluate participants’ digital therapeutic use patterns of PEAR-008 compared to reSET-O	Session duration; number of lessons and chapters completed; number of completed self-report assessments; response to notifications	
	To evaluate the impact of PEAR-008 compared to reSET-O on change in psychiatric symptom severity between baseline and follow-up	Depression symptoms with Patient Health Questionnaire–8; Anxiety symptoms with Generalized Anxiety Disorder–7	
	To evaluate the impact of PEAR-008 compared to reSET-O on change in recovery capital and resilience between baseline and follow-up	Brief Assessment of Recovery Capital–10; Connor-Davidson Resilience Scale–10	
	To evaluate participant motivation and satisfaction over time with PEAR-008 compared to reSET-O	Participant surveys administered at baseline, week 4, week 8, and week 12; participant interview at follow-up	
	To evaluate a more global measure of engagement by combining data from secondary endpoints 3-6	A multivariate analysis of variance combining data from secondary endpoints 3-6	
**Exploratory**			
	To evaluate the association between engagement with PEAR-008 compared to reSET-O and treatment outcomes (abstinence and retention)	Daily and weekly use patterns; saliva (or urine drug screen for AREF^a^ participants only) and self-report; time to dropout	
	To evaluate skills acquisition at week 4 and week 8 of PEAR-008 compared to reSET-O	Cognitive Behavioral Therapy Skills Questionnaire	
	To evaluate medication adherence of PEAR-008 compared to reSET-O	Saliva drug screen (or urine drug screen, for AREF participants only)	

^a^AREF: Addiction Research and Education Foundation.

### Assessments

#### Demographics

Demographic information will be collected at the baseline session. Variables to be collected will include age at time of consent, sex assigned at birth, race and predominant self-reported ethnicity, level of education, marital status, employment status, occupation, and legal status.

#### Medical and Medication History

Medical history and current medical conditions, with current or prior treatment received, such as mental health disorders, hepatitis C virus, human immunodeficiency virus, and chronic pain syndromes, will be collected at the baseline session. Participant-reported history of substance use will be collected for the following substance categories: opioids, cocaine, stimulants (other than cocaine), alcohol, marijuana, benzodiazepines, other. Data to be collected regarding each substance include age at onset of use, number of years used regularly, amount used, type used (eg, pill vs powder), route of administration, history of overdose, and longest period of abstinence. Participant-reported history of substance use treatment will be collected, including present and prior treatment, type of treatment facility, number of treatment episodes, and current recovery activities. Nicotine use history will also be collected, including type, route, quantity, duration of use, and prior use. Medication history will include the name, dose, frequency, start and stop dates of the medication, and the indication for use. Prior or current medication for substance use disorder and opioid use disorder will also be recorded.

### Clinical Assessments

#### Abstinence From Opioids and Buprenorphine Adherence

Participant self-report of substance use and medication adherence will be collected within the reSET-O and PEAR-008 apps. In PEAR-008, participants are prompted to report use or abstinence each time they open the app as part of the check-in feature. In reSET-O, participants are prompted to report substance use or abstinence every 72 hours and can choose to self-initiate responses anytime.

Timeline followback will be completed at each virtual study session to assess patient-reported substance use since the time of the last assessment. The timeline followback is a validated calendar-based assessment used to obtain self-reports of amount and frequency of substance use retrospectively, using memory aids to enhance recall (eg, patterns of use, key dates).

Participants will self-administer a 12-panel saliva drug test during video sessions with study staff, who can assist in proper administration in addition to providing high-fidelity confirmatory testing. The 12-panel tests detect the presence of the following drugs: amphetamines, cocaine, cannabis, opioids, methamphetamine, barbiturates, benzodiazepines, buprenorphine, oxycodone, methadone, fentanyl, and alcohol. Saliva drug testing will be performed at the baseline session, once weekly during weeks 1-8 of the treatment phase and at the week 12 follow-up session. Participants will receive drug screening equipment regularly by mail.

When available, results of urine drug screening collected as part of routine care at participating treatment centers will also be used for participants recruited by AREF. All urine drug screen data are collected for clinical purposes and not as a study procedure. Urine drug screen results will be exported from the electronic medical record for inclusion in the study database. These data will be used to evaluate buprenorphine adherence and drug use. Results from additional urine drug screen confirmatory analyses for buprenorphine and norbuprenorphine may be available for some participants. When available, these data will be used to evaluate buprenorphine adherence.

#### Psychiatric Symptoms

Individuals with substance use disorders often experience psychiatric comorbidities, such as depression and anxiety. To evaluate the change in severity of co-occurring depression and anxiety symptoms, Patient Health Questionnaire–8 and Generalized Anxiety Disorder Questionnaire assessments will be delivered at baseline, week 4, week 8, and week 12.

The Patient Health Questionnaire–8 is an 8-item multipurpose instrument for screening and monitoring changes in depression [30]. The Generalized Anxiety Disorder Questionnaire–7 is a 7-item questionnaire for screening and monitoring changes in symptoms related to generalized anxiety disorder [31].

#### Recovery Status, Resilience, and Cognitive Behavioral Therapy Skills

The Substance Abuse and Mental Health Services Administration developed a working definition of recovery that includes 4 major dimensions: Health, Home, Purpose, and Community [32]. This definition highlights the importance of including measures of recovery across these dimensions in addition to substance use outcomes such as abstinence.

The Brief Assessment of Recovery Capital–10 is a 10-item assessment that will be used to measure participants’ level of recovery capital [33]. Recovery capital consists of a variety of resources and strengths that patients can use to support their recovery. The 4 major dimensions of recovery are thought to be strengthened as an individual builds recovery capital and may indicate an individual’s likelihood of remaining in remission [34].

The Connor-Davidson Resilience Scale–10 is a 10-item self-rating scale developed to assess resilience [35]. The measure is an abbreviated version of the 25-item version and was established on the basis of a factor analysis in a community sample. The questionnaire asks individuals to indicate how much they agree with a series of statements on a 5-point Likert scale. Resilience is a multidimensional trait characterized by an individual’s capacity to maintain normal functioning and resist the development of psychiatric symptoms and disorders in response to stress and adversity. The Connor-Davidson Resilience Scale will be used to evaluate the malleability and stability of trait resilience over courses of treatment with reSET-O and PEAR-008.

The Cognitive Behavioral Therapy Skills Questionnaire is a 16-item assessment that measures the use and acquisition of cognitive behavioral therapy skills during treatment [36] and is a validated measure of behavioral activation and cognitive restructuring. Development of skills that support behavior change is a key goal of cognitive behavioral therapy. This scale will be used to assess the development of cognitive behavioral therapy skills and whether there is a difference in skill development between individuals treated with reSET-O versus those treated with PEAR-008.

These scales will be administered at baseline, week 4, week 8, and week 12.

#### COVID-19 Impact

Two exploratory assessments will be used to evaluate the impact of the COVID pandemic on participants. The CAIR Pandemic Impact Questionnaire [37] is a 5-item assessment that measures how the COVID-19 pandemic is impacting participants, using a 5-point Likert scale to evaluate whether the respondent has experienced any growth changes related to COVID-19 in the past 2 weeks. A modified version of the Coronavirus Perinatal Experiences–Impact Survey asks participants how they are coping with stress related to COVID-19 from a list. The respondent is provided with a list of coping strategies and asked to select all strategies they have employed. Both assessments were selected from the PhenX Toolkit [38] and will be administered at baseline, week 4, week 8, and week 12.

#### Participant Motivation and Satisfaction

Participant motivation and satisfaction will be evaluated via surveys and qualitative interviews. Surveys will be administered at baseline, week 4, week 8, and week 12, with a range of questions designed to evaluate participant motivation (baseline only) and satisfaction with their assigned intervention (eg, ease of use, relevance, satisfaction).

A subset of participants (approximately 10 per treatment arm) will be asked to participate in a 1-on-1 qualitative interview to evaluate their respective experiences with each therapeutic, particularly ease of use and acceptability. Interviews will last approximately 60 minutes, be conducted by study staff, and will be performed remotely via video or phone. Interview transcripts will be coded and analyzed using grounded theory methodology to identify key themes. An inductive, open coding approach will be used to assign emerging categories. Emerging categories will be grouped to arrive at high-level themes during axial coding, and their properties and dimensions will be identified and described in a codebook.

### Data Management, Study Oversight, and Monitoring

Oversight of data management, including data collection, storage, export, security, tracking, data analysis, and quality assurance will be the responsibility of the study monitor designated by the study sponsor. Trial data will be managed with an electronic data capture system (Captivate, ClinCapture). Sites have access to this software as does the sponsor data monitor. Data collected by PEAR-008 and reSET-O are stored in the cloud. A data and safety monitoring board will provide additional oversight regarding the safety of study participants.

### Adverse Events and Safety Monitoring

The investigator or designee and research site staff will be responsible for the detection, documentation, classification, reporting, and follow-up of events that meet the definition of an adverse event or serious adverse event. Spontaneously reported or observed adverse events will be recorded throughout the study from the time of consent until the end of the last study visit. Adverse events will be elicited using a nonleading question at designated time points. Regardless of seriousness, intensity, or presumed relationship to reSET-O or PEAR-008, all adverse events will be recorded. The site investigator will monitor the occurrence of adverse events during the study.

Adverse events and serious adverse events will be collected and reported using the methods and definitions of the Office for Human Research Protections and National Institutes of Health (NIH) requirements for human participant protection. The investigator or designee is responsible for making an assessment as to the seriousness, intensity, causality, and outcome of an adverse event. The investigator will determine causality as related, possible, unlikely, or unrelated to reSET-O or PEAR-008.

### Confidentiality

Procedures to assure confidentiality will be strictly observed. All participant personal information will be kept confidential and will not be released without written permission, except as required by law. All study information will be kept separately from identifying information on consent forms and locator forms.

Data collected by the reSET-O and PEAR-008 system are securely transferred using industry standard encryption to a cloud-based infrastructure that serves and communicates with the patient facing mobile app; the backend services contain all data and analytics for reSET-O, PEAR-008, and clients (participants and clinicians). All data stored by the device are hosted and stored with a cloud-computing service (Amazon Web Services), which follows a variety of internationally recognized security standards, such as National Institute of Standards and Technology SP800-53 [39] and Health Insurance Portability and Accountability Act [40]. All patient information is automatically encrypted when it is entered into the system, which allows for secure data transfer (from patient device to clinician device) and storage.

In accordance with the 21st Century Cures Act [41], all ongoing or new research funded by NIH as of December 13, 2016 that collects or uses identifiable, sensitive information is automatically issued a Certificate of Confidentiality, which protects participants against disclosure of any sensitive information or illicit behavior (eg, drug use).

### Statistical Analysis

All statistical methods will be consistent with the International Conference on Harmonization E9 Guidance [42]. Data will be summarized by treatment group. For baseline, safety, and efficacy outputs a total population will combine both groups. Where appropriate, the data will be summarized by session in addition to treatment group. Baseline, demographic, and efficacy output data will be summarized by intended treatment. Safety output will be summarized by the treatment received.

Every effort will be made to obtain required data at each scheduled evaluation. Missing data will not be imputed. Sensitivity analyses incorporating various imputation assumptions may be performed if missing data exceed 5% of the total possible observations.

Descriptive statistics will be used to describe the population of study participants at the beginning of the study (mean, standard deviation, minimum, 25th percentile, median, 75th percentile, and maximum for continuous data; frequencies and percentages for categorical data). Differences in continuous variables will be summarized with Hedges *g* effect size; differences in categorical variables will be summarized with odds ratios.

### Endpoints

#### Primary Endpoint Analysis

Engagement with PEAR-008 and reSET-O will be defined as the number of active sessions per week. A session is defined as a set of in-app events with the same session ID. An active session is any session that contains some active participation in the app such as navigating to a different screen, engaging with a learning module, or responding to a notification. This endpoint was selected to allow a 1:1 comparison of content delivery method (eg, brief chapters vs longer lessons). The number of active sessions per week will be evaluated with a repeated measures mixed model of the form: *Number of Sessions in a Week = Week + Treatment + Week × Treatment + Subject Error + Random Error*. To evaluate the impact of treatment, this repeated measures mixed model will be compared to another of the form: *Number of Sessions in a Week = Week + Subject Error + Random Error* with a likelihood ratio test. The likelihood ratio test will be evaluated using model fit to maximize the log-likelihood.

#### Secondary Endpoint Analysis

Abstinence will be self-reported by patients via timeline followback and assessed as the absence of opioids (except buprenorphine) on saliva drug screens. Data on abstinence (binary yes and no) will be evaluated with the 9 possible saliva drug screens and urine drug screen and timeline followback data at weeks 1-8 and week 12. Differences between the treatment arms in abstinence will be assessed with a generalized estimating equation analysis using the binomial abstinence outcome as the dependent variable, assessment time, treatment, and the interaction between assessment time and treatment as fixed effects and subject as a random factor. This generalized estimating equation will be compared to a second generalized estimating equation without a treatment term using a likelihood ratio test to evaluate the impact of treatment. A treatment responder analysis will also be conducted, with a treatment responder defined as someone with ≥80% of all saliva drug screens and urine drug screens and timeline followback reports negative for all nonprescribed opioids over the course of the trial.

Treatment retention will be measured as time to dropout (number of days from baseline to last face to face contact) and analyzed using the Kaplan Meier method followed by a log rank test to compare treatment groups.

Patient assessments of recovery capital (Brief Assessment of Recovery Capital–10) resilience (Connor-Davidson Resilience Scale–10) and severity of co-occurring psychiatric symptoms (Patient Health Questionnaire–8 and Generalized Anxiety Disorder Questionnaire–7) will be evaluated with a repeated measures mixed model in a manner consistent with the primary endpoint.

Participants’ use patterns with PEAR-008 and reSET-O will include total time engaged, number of lessons and chapters completed, number of lessons and chapters repeated, number of completed self-report assessments, and response to notifications. Descriptive statistics of each treatment arm will be summarized and differences between PEAR-008 and reSET-O will be assessed with a *t* test (or its nonparametric alternative if the distributional assumptions are violated).

Treatment motivation and satisfaction will be evaluated based on individual participants’ rating of interest in using (baseline only), ease of use, satisfaction, perceived helpfulness, and likelihood of recommending reSET-O or PEAR-008. Descriptive statistics (mean, median, standard deviation) will be performed on Likert scale and multiple choice items that assess user satisfaction and attitudes about the patient mobile app. Analyses will be conducted on data collected at each assessment point throughout the study, ratings over the course of the study, and for overall user satisfaction as measured at the end of the study.

#### Exploratory Endpoint Analysis

Data on abstinence (binary—yes and no) will be evaluated at 9 saliva drug screen and timeline followback time points at weeks 1-8 and week 12. The association between abstinence and engagement will be evaluated using a generalized estimating equation model. A given week will be considered negative if a participant has no indication of opioid use on the saliva drug screens or timeline followback. Missing data will be ignored if saliva drug screens or a timeline followback is available for a given time point. In the case where saliva drug screens and timeline followback methods yield data that do not agree, the week will be considered positive.

The relationship between engagement and retention will be assessed using a Cox proportional hazards model with time to dropout as the dependent variable and the number of active sessions as an independent variable. This analysis will be performed separately for each treatment arm.

Patient assessment of skill acquisition (Cognitive Behavioral Therapy Skills Questionnaire) will be evaluated with repeated measures mixed model analysis consistent with the primary endpoint. Results will be presented for the total Cognitive Behavioral Therapy Skills Questionnaire score, the Behavioral Activation Score, and the Cognitive Restructuring score.

Medication adherence will be evaluated using saliva drug screens and, when possible, confirmed by urine drug screen (positive result for buprenorphine and norbuprenorphine). Differences between the treatment arms in medication adherence will be assessed with a generalized estimating equation analysis consistent with the abstinence analysis.

### Ethics Approval

This study protocol has been reviewed and approved by the New York State Psychiatric Institute Institutional Review Board. The study is registered on ClinicalTrials.gov (NCT04542642).

## Results

Recruitment for this study was active as of February 2021 and will continue until the projected sample size is met.

## Discussion

While initially designed as a standard, site-based clinical trial, this study was redesigned as a decentralized study to circumvent challenges in conducting in-person visits that arose as a result of the COVID-19 pandemic. Standard in-person procedures for this patient population that were planned for the study, such as initiation of buprenorphine medication in-clinic or on-site urine drug screening, were no longer feasible. The decentralized study model aligns with a shift in health care delivery observed during the pandemic, as many substance use disorder treatment providers were able to transition to telemedicine for care delivery, including initiation onto buprenorphine and maintenance treatment [43,44]. Digital therapeutics lend themselves to remote therapy delivery models, presenting a unique opportunity to evaluate their use along with existing technology and tools such as videoconferencing, electronic signatures for documenting consent, and video-observed, self-administered saliva drug test kits.

Several challenges arose with the shift in study design. One of the sites, a research clinic, typically manages buprenorphine medication during studies of this patient population. Without in-clinic participant visits, it became necessary for the site to confirm that participants were receiving buprenorphine under the care of a licensed prescriber. This was accomplished by obtaining release of medical information waivers from prospective participants, allowing the site to reach out directly to the individual’s treatment provider for confirmation of eligibility. This pivot allowed the research clinic to recruit a broader geographic sample of individuals than is typical for similar studies [45] conducted on-site. Another challenge was ensuring that all assessments could be conducted remotely. While most self-report assessments were easy to transition to virtual delivery, it is challenging to conduct urine drug screening virtually. Saliva drug screening kits were selected as an alternative that allowed participants to self-administer the test during video sessions and under supervision by study staff. Finally, participant recruitment methods shifted toward a strategy focused on digital media and treatment programs using telemedicine.

Several questions about decentralized studies in this patient population will be addressed by this study. For example, it is not yet clear whether it will be easier to recruit and retain individuals with opioid use disorder in a virtual study than in a standard face-to-face study. Providing a more convenient mechanism for conducting study sessions may increase retention, which can be challenging in this patient population. Evaluating the utility of self-administered saliva drug screening may also be beneficial to other investigators who are considering this method of evaluating substance use. Challenges that arise over the course of the study will help elucidate the limitations of this study design. This example of a decentralized clinical trial may provide a useful model for conducting future virtual studies with people with opioid use disorder.

## Abbreviations

AREF: Addiction Research and Education Foundation
FDA: US Food and Drug Administration
NIH: National Institutes of Health
STARS: Substance Treatment and Research Services
